# Odds of Developing Overweight or Obesity Increase with Height Decile in Tall Healthy Weight Kindergarteners: Longitudinal Results from the West Virginia Coronary Artery Risk Detection In Appalachian Communities (WV CARDIAC) Project

**DOI:** 10.1016/j.jpedcp.2025.200197

**Published:** 2025-12-08

**Authors:** Maria A. Serrat, Eloise Elliott, Lee A. Pyles, Christa L. Lilly

**Affiliations:** 1 Department of Biomedical Sciences, Joan C. Edwards School of Medicine, Marshall University, Huntington, WV; 2 School of Sports Sciences, West Virginia University, Morgantown, WV; 3 Department of Pediatrics, Division of Cardiology, West Virginia University, Morgantown, WV; 4 Department of Epidemiology and Biostatistics, West Virginia University, Morgantown, WV

**Keywords:** BMI, tall stature, rapid growth, obesity risk detection

## Abstract

**Objective:**

Childhood obesity is a strong predictor of adult obesity and chronic health complications. Prevention initiatives are most successful when implemented before excess weight gain, but tools to screen healthy weight children for obesity risk are lacking. Laboratory studies suggest that tall stature could be an early indicator of later obesity. We sought to test the hypothesis that kindergarten height is associated with body mass index (BMI) trajectories through second and fifth grade in Appalachian school children.

**Study design:**

Children with BMI data in kindergarten and second or fifth grade (n = 9059) were obtained from the West Virginia Coronary Artery Risk Detection In Appalachian Communities (WV CARDIAC) Project. BMI percentile trajectories were calculated using high performance mixed modeling to save the best linear unbiased predictor random effect slopes. Subset analyses were performed on children with healthy weight BMI in kindergarten (n = 5786). Linear mixed models on BMI trajectories and logistic regression models on children in the second or fifth grade with overweight/obesity were conducted.

**Results:**

After we controlled for sex, age, and kindergarten BMI, kindergarten height percentile was associated with rapid BMI trajectories (linear mixed models Est. = 0.01, *P* < .0001). For children in the healthy BMI weight category, the odds of developing overweight/obesity increased with each height decile. The tallest children in kindergarten (top decile) had 3 times the odds of developing overweight/obesity compared with the first decile (OR 2.97, 95% CI 2.15-4.10).

**Conclusions:**

Obesity-prevention initiatives could greatly benefit from considering tall stature and rapid growth velocity in early childhood as potential indicators of obesity risk, even if children fit within a healthy BMI category.

## Graphical abstract

**Figure:**
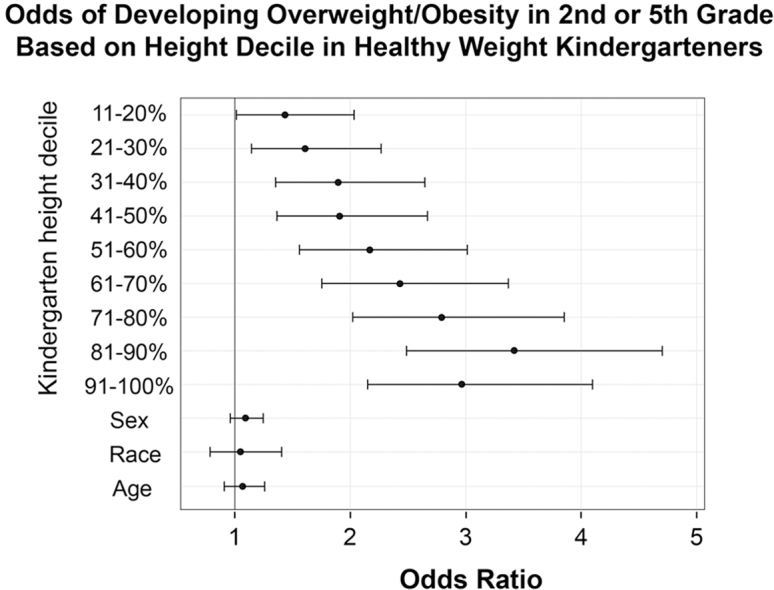

Childhood obesity is a strong predictor of adult obesity^1^, ^2^, ^3^ and chronic comorbidities such as type 2 diabetes, hypertension, hypertriglyceridemia, fatty liver, sleep apnea, and cancer.^4^, ^5^, ^6^, ^7^, ^8^, ^9^, ^10^, ^11^, ^12^ Although the underpinnings of obesity are multifaceted, a primary modifiable contributor is diet,^13^, ^14^, ^15^, ^16^, ^17^ which can exacerbate a genetic predisposition to weight gain.^18^ Prevention strategies focused on diet in early childhood are considered key for promoting long-term healthy lifestyle habits,^19^^,^^20^ but timing is critical. Programs to prevent obesity are most successful when implemented in young children before they gain excess weight.^21^ After the onset of obesity, success rates of dietary interventions for weight loss and reversal of health problems can be low.^6^^,^^19^^,^^20^^,^^22^^,^^23^ There is a critical need for reliable screening tools to identify early obesity risk in young children with healthy weight.^15^^,^^24^, ^25^, ^26^, ^27^, ^28^

Rapid linear growth is a hallmark feature of obesity.^29^^,^^30^ Children with obesity are only temporarily taller, however, because they reach maturity and attain final adult height sooner than their peers.^31^, ^32^, ^33^, ^34^ Evidence from a controlled laboratory study suggests that a steep growth trajectory can precede the development of obesity. Using mice that were weaned onto a high-fat diet, Machnicki et al (along with the first author here—Serrat) reported that the animals exhibited accelerated bone elongation before they had a significant increase in weight gain.^35^ Retrospective studies of adolescent humans with obesity have shown that rapid growth and tall stature also were evident in these children before their diagnosis of obesity.^32^^,^^36^, ^37^, ^38^, ^39^, ^40^, ^41^

Accelerated linear growth could be a key metric in identifying children at risk for obesity, which is traditionally defined using body mass index (BMI, kg/m^2^).^15^ A healthy weight BMI for children ranges from the fifth to 85th percentile on age- and sex-specific reference charts, whereas overweight is between the 85th and 95th percentiles, and obesity is a BMI ≥95th percentile.^42^ Because children with excess adiposity are also tall for their age,^29^^,^^30^ their increased height can offset excess weight in the BMI formula (kg/m^2^), leading to a lower BMI and delay in flagging a child for obesity risk. No guidance currently exists for monitoring obesity risk in children within the healthy weight BMI category, and thus the opportunity for an early intervention is often missed by screening based on BMI alone.

The goal of our study was to determine whether tall stature could be used as an early indicator of obesity risk, irrespective of BMI category. We tested the hypothesis that height percentiles in kindergarten are associated with rapid BMI percentile trajectories through second and fifth grade in a sample of Appalachian school children.

## Methods

The West Virginia Coronary Artery Risk Detection In Appalachian Communities (WV CARDIAC) Project started as a small school-based cardiovascular disease surveillance program piloted in 3 rural West Virginia counties in 1998.^43^ It expanded to a multidimensional screening, research, and intervention effort involving all 55 West Virginia counties and more than 480 schools that has since provided information to participating families, schools, health care providers, and the state and nation about chronic illnesses including hypertension, hyperlipidemia, obesity, and other health-related conditions in children.^12^^,^^44^, ^45^, ^46^ Aggregate data, along with detailed study methodology, are published.^5^^,^^43^^,^^44^^,^^47^, ^48^, ^49^, ^50^, ^51^, ^52^, ^53^ All WV CARDIAC assessment protocols were approved by the West Virginia University Institutional Review Board for the Protection of Human Subjects.

**Figure 1:**
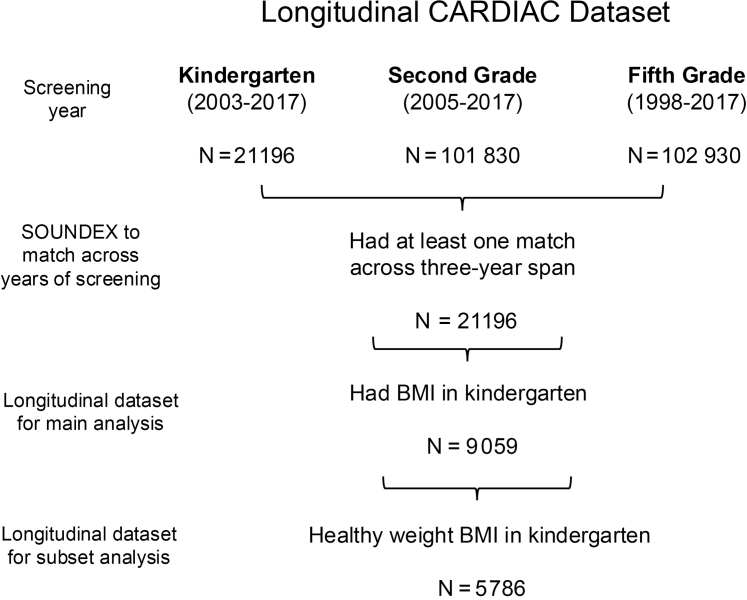
Schematic of criteria used to obtain datasets in the study.

For this study, secondary data analyses were conducted on 3 cross-sectional WV CARDIAC datasets collected between 1998 and 2017 (Figure 1). Children were screened in kindergarten (n = 21 196; 2003-2017), second (n = 101 830; 2005-2017), and fifth (n = 102 930; 1998-2017) grades. Students were matched across years of screening using SOUNDEX^54^ to account for misspellings for first and last name and combining those values with the date of birth to provide a unique identifier that could be directly matched across the 3 datasets. This resulted in 21 696 unique students having at least 1 match across a 3-year span. Of those, 9059 had a BMI measurement in kindergarten and were included in this study.

### Height and Weight Measurements

BMI percentiles were calculated at each year from each child's height (centimeters) and weight (kilogram), which were measured using the SECA Road Rod stadiometer (7800/200 cm) and the SECA 840 Personal Digital Scale. Students were asked to remove shoes before height and weight measurements. These measurement were used to determine the child's BMI, BMI percentile (adjusting for age and sex), and height percentile (adjusting for age and sex) calculated using CDC Epi Info version 3.5.4 software.^55^ For some analyses, height percentiles were categorized into deciles. BMI percentiles were used to partition children into categories that included underweight, healthy weight, overweight, and obesity according to CDC guidelines.^56^

### BMI Percentile Trajectories

BMI percentile trajectories over time were calculated using BMI percentiles. First, a best-fitting linear mixed model was fitted with the BMI percentile as the dependent variable and student grade as the predictor. The grade (as a proxy of time) was included as a random slope and intercept. The SAS procedure high performance mixed modeling (HPMIXED) was then used to save the best linear unbiased predictors from the random intercept and slopes. These slopes, or BMI trajectories, were then merged back into the dataset.

### Demographics and Family History

Demographics included the kindergarten age at screening in years, calculated from the reported birth date and screening date. Sex was reported by the parent as male or female. Race included options for Black, White, Asian, Hispanic, biracial, and other on the screening form. Race was recoded to White or non-White for this study because of the low representation of other race categories. Because the screening questionnaire focused solely on family history of cardiovascular disease, there were no exclusion criteria in this study due to family history of endocrine-related disorders because those data were not collected (see Discussion section for limitations).

### Statistical Analyses

SAS, version 9.4 (SAS Institute) was used for all analyses. After BMI trajectories were calculated, only children with data in kindergarten and at least 1 measurement in second or fifth grade were included (n = 9059). Descriptive statistics included means and SDs for continuous variables, and frequencies and valid percentages for categorical variables. Linear mixed models with REML estimation were run with the BMI trajectories as the outcome variable, kindergarten height percentile as the primary exposure, and age, sex, dichotomized race, and BMI category in kindergarten as covariates. The interaction of height percentile with BMI category also was included in an additional model. Stratification was conducted by BMI category to better understand the differences in growth over time. Finally, we examined a logistic regression model on a subset of normal weight children in kindergarten. We examined whether their height deciles increased the odds of developing an overweight or obese category either in second or fifth grade, again controlling for age, sex, and race. Estimates were exponentiated and presented as ORs with Wald CIs. For comparison, we also examined children with high BMI in kindergarten to assess whether height was among the factors associated with remaining in a high BMI category in second or fifth grade by running another logistic regression model on a subset of children classified with overweight or obesity in kindergarten (n = 3030).

## Results

### Demographics

Demographics are listed in Table I. Of the 9059 included kindergarten children, 5469 (60.4%) had another measurement in second grade only, 1499 (16.5%) had another measurement in fifth grade only, and 2091 (23.1%) had measurements in both second and fifth grade. Approximately one-half of the participants were male (n = 4490; 49.6%) and the majority with a reported race were White (n = 7546 of 8049; 93.8%). More than one-half had a BMI categorized as healthy weight in kindergarten (n = 5816; 64.2%) and were on average 5.9 (SD = 0.4) years old when they were screened.

**Table I:** Demographic and descriptive statistics

Variables	Total No.	Frequency	Valid percentage
Sex	9059		
Male		4490	49.6
Female		4569	50.4
Kindergarten BMI category	9059		
Underweight		195	2.1
Normal weight		5816	64.2
Overweight		1537	17
Obese		1511	16.7
Second-grade BMI category	7515		
Underweight		146	2
Normal weight		4556	60.6
Overweight		1232	16.4
Obese		1581	21.0
Missing	1544		
Fifth-grade BMI category	3564		
Underweight		76	2.1
Normal weight		1837	51.5
Overweight		664	18.6
Obese		987	27.8
Missing	5495		
Race	8049		
Black		121	1.5
White		7546	93.8
Asian		57	0.7
Hispanic		46	0.6
Bi-racial		244	3.0
Other		35	0.4
Missing	1010		
Matches to kindergarten	9059		
Second grade only		5469	60.4
Fifth grade only		1499	16.6
Both second and fifth		2091	23.0

Variables	Total No.	Mean	SD	Min, max
Age (kindergarten)	9059			
years		5.9	0.4	4.8, 8.0
Height (kindergarten)	9059			
Percentile		57.3	28.6	0.01, 100.00
BMI percentile trajectory	9059			
BLUP slope		−7.3	8.4	0.7, 1.5

*BLUP*, best linear unbiased predictor.

### BMI Percentile Trajectories

Height percentile in kindergarten was significantly associated with increasing BMI percentile trajectories over time (est. = 0.01, *P* < .0001), even after controlling for child sex, age, race, and BMI category in kindergarten (Table II, model 1). When we examined the interactions (Table II, model 2), the main effect of height percentile was maintained even with interaction inclusion (Est. = 0.01, *P* < .0001). Underweight (relative to healthy weight) saw the most gains over time for taller children (interaction est. = 0.02, *P* < .0001); whereas children who were already categorized with overweight or obesity in kindergarten had slower BMI percentile trajectory for taller children (interaction est. obese −0.01, *P* = .005; overweight est. = −0.01, *P* = .003).

**Table II:** Fixed effects of the linear mixed models, showing associations with the outcome of BMI trajectory over time (BLUP slopes) and predictors in kindergarten (n = 9059).

Models/Effect	Estimate	SE	t-value	*P* value
Model 1				
Intercept	−0.46	0.23	−2.02	.0435
Child sex				
Female	−0.12	0.03	−3.83	.0001
Male (ref)				
Race				
Other	0.02	0.07	0.27	.7876
Not reported	0.05	0.05	1.09	.2753
White (ref)				
Age				
Years	0.15	0.04	3.63	.0003
BMI category				
Obese	−0.41	0.04	−9.27	<.0001
Overweight	−0.37	0.04	−8.94	<.0001
Normal weight (ref)				
Underweight	1.09	0.11	10.39	<.0001
Height				
Percentile	0.01	0.001	13.87	<.0001
Model 2				
Intercept	−0.52	0.23	−2.25	.0244
Child sex				
Female	−0.12	0.03	−3.85	.0001
Male (ref)				
Race				
Other	0.02	0.07	0.24	.8084
Not reported	0.05	0.05	1.10	.2721
White (ref)				
Age				
Years	0.15	0.04	3.88	.0001
BMI category				
Obese	−0.07	0.13	−0.58	.5605
Overweight	−0.10	0.10	−1.06	.2909
Normal weight (ref)				
Underweight	0.10	0.22	0.46	.6243
Height				
Percentile	0.01	0.001	12.73	<.0001
Height∗BMI category				
Obese	−0.01	0.002	−2.83	.0047
Overweight	−0.01	0.002	−3.01	.0026
Normal weight (ref)				
Underweight	0.02	0.003	5.19	<.0001

Model 1 includes the main effect of height percentile with covariates of age, race, sex, and BMI category. Model 2 additionally includes interaction of height percentile with BMI category.

When stratified by kindergarten BMI category, Table III demonstrates similar results to the interaction model. Height percentile is statistically associated with increased BMI trajectory over time (from kindergarten to fifth grade) regardless of BMI category after controlling for sex, race, and age in kindergarten (underweight height percentile Est. = 0.03, *P* < .0001; healthy weight est. = 0.009, *P* < .0001; overweight est. = 0.004, *P* < .0001, obesity est. = 0.004, *P* < .0001).

**Table III:** Fixed effects of the linear mixed models, showing associations with the child's BMI trajectory over time (BLUP slopes) and predictors in kindergarten, stratified by BMI category in kindergarten

Effect	Underweight (n = 195)			Healthy weight (n = 5816)			Overweight (n = 1537)			Obese (n = 1511)		
	Est.	SE	*P* value	Est.	SE	*P* value	Est.	SE	*P* value	Est.	SE	*P* value
Intercept	−0.93	2.11	.6607	−0.53	0.33	.1088	−0.90	0.38	.0165	−0.24	0.23	.3001
Child sex												
Female	−0.16	0.28	.5675	−0.19	0.04	<.0001	0.06	0.05	.2567	0.01	0.03	.8209
Male (ref)												
Race												
Other	−0.06	0.58	.9186	0.02	0.10	.8311	0.01	0.10	.9401	0.01	0.06	.8368
Not reported	−0.27	0.35	.4360	0.08	0.07	.2596	0.08	0.08	.3534	0.01	0.05	.9021
White (ref)												
Age												
years	0.25	0.35	.4768	0.15	0.05	.0045	0.18	0.06	.0041	0.08	0.04	.0374
Height												
Percentile	0.03	0.005	<.0001	0.009	0.001	<.0001	0.004	0.001	<.0001	0.004	0.001	<.0001

### Odds of Developing Overweight or Obesity on the Basis of Height Decile in Children with Healthy Weight

The main subset analysis included only children who were categorized with healthy weight BMI in kindergarten (n = 5816) because we were most interested in the impact of stature (analyzed by height deciles) on the development of overweight or obesity. Of those, 5786 were included in a final logistic regression model (n = 30 had height or weight missing in second or fifth grade). We then divided those 5786 kindergarteners in the healthy weight category into deciles on the basis of height to determine the odds of taller children developing overweight or obesity by the second or fifth grade. ORs for each height decile are calculated relative to the lowest height decile.

**Figure 2:**
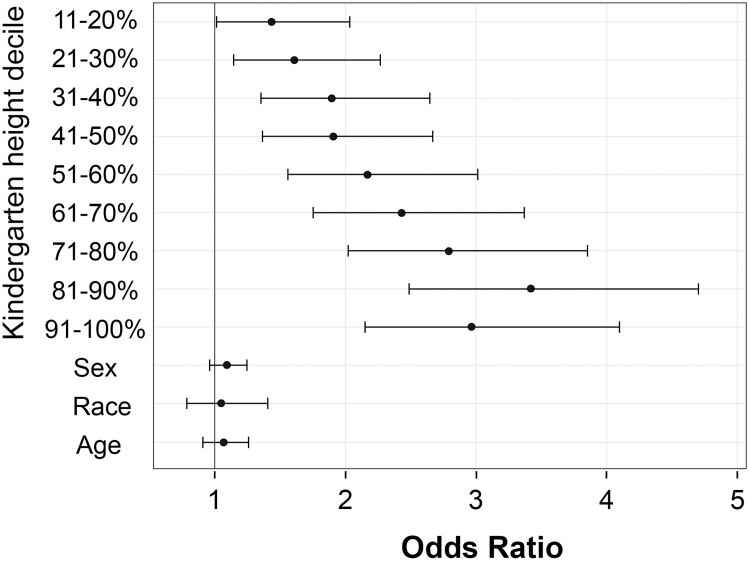
Odds of developing overweight or obesity in second or fifth grade on the basis of height decile in kindergarteners of healthy weight (n = 5786). The ORs, which increased progressively with height, were significant for all height deciles. The tallest children in kindergarten (top 3 deciles) had 3 times the odds of developing overweight or obesity when compared with the children of shortest stature. There was no effect of sex (male vs female), race (White vs non-White) or age on the later development of overweight or obesity in this cohort. ORs are plotted with 95% Wald CIs.

Figure 2 shows that, relative to the children who were shortest in kindergarten, the odds of developing overweight or obesity increased progressively with each increasing height decile. The children who were tallest in kindergarten (80th height percentile and above) had 3 times the odds of developing overweight or obesity when compared with the children who were shortest (below 10th percentile). The 95% CIs do not cross 1, which indicates that these results are significant for all height deciles (Table IV, Figure 2). Although height in kindergarten was a significant factor in the later development of overweight or obesity, there was no effect of sex (male vs female), race (White vs non-White), or age. Notably, relative to the lowest decile, we see that the tallest children in the top 3 deciles have significantly increased odds (2.79-3.42) of developing overweight or obesity by second or fifth grade (Table IV, Figure 2).

**Table IV:** Logistic regression of developing overweight/obesity on the basis of height decile in healthy weight kindergartners, n = 5786

Effects	Est.	SE	χ^2^	*P* value	OR	Lower 95% Cl	Upper 95% CI
Intercept	−1.84	0.49	13.97	.0002			
Height percentile decile (ref 0-10%)							
11%-20%	−0.35	0.11	10.20	.0014	1.44	1.01	2.03
21%-30%	−0.24	0.11	4.96	.0259	1.61	1.14	2.27
31%-40%	−0.08	0.11	0.55	.4594	1.89	1.35	2.65
41%-50%	−0.07	0.11	0.43	.5118	1.91	1.36	2.67
51%-60%	0.06	0.10	0.39	.5313	2.17	1.56	3.01
61%-70%	0.18	0.10	3.39	.0654	2.43	1.75	3.37
71%-80%	0.32	0.10	11.58	.0007	2.79	2.02	3.85
81%-90%	0.52	0.10	33.80	<.0001	3.42	2.49	4.70
91%-100%	0.38	0.10	16.74	<.0001	2.97	2.15	4.10
Child sex							
Female	0.05	0.03	1.83	.1764	1.09	0.96	1.25
Male (ref)							
Race							
Other	0.13	0.10	1.57	.2106	1.05	0.79	1.41
Not reported	−0.21	0.09	5.97	.0146	0.75	0.61	0.93
White (ref)							
Age							
Years	0.07	0.08	0.64	.4248	1.07	0.91	1.26

### Maintenance of Overweight or Obesity in Children with High BMI in Kindergarten

We also examined a subset of children who were classified with overweight or obesity in kindergarten to assess whether height was among the factors associated with remaining in a high BMI category. Of the 3030 children with overweight or obesity BMI in kindergarten, the majority (n = 2555, or 84.3%) remained in the overweight or obesity BMI category in second or fifth grade, whereas 475 (15.7%) returned to a normal weight BMI category. This finding was more pronounced for those who had a BMI greater than 95th percentile in kindergarten (n = 1498), of whom only n = 59 (3.9%) returned to a normal weight BMI category by second or fifth grade. We then ran a logistic regression on this high BMI group (n = 3030) to identify potential factors in maintaining overweight or obesity status.

Logistic regression performed on the subset of children classified with overweight or obesity in kindergarten revealed similar findings to those in the normal-weight category. Although height in kindergarten was a significant factor in maintaining overweight or obesity status, there was no effect of race (White vs non-White; *P* = .57). However, there was a significant effect of age (aOR 1.91; 95% CI 1.48-2.47; *P* < .0001), with children who were older at increased odds of maintaining overweight or obesity status at a later grade. There was a slight effect of sex (aOR 0.81, 95% CI 0.66-0.99; *P* = .04), with girls at slightly lower odds of maintaining overweight or obesity status. Notably, relative to the lowest height decile, all height deciles were at significantly increased odds (2.06-7.99) of maintaining overweight or obesity in second or fifth grade, and this difference was especially pronounced for children in the top 2 height deciles in kindergarten.

## Discussion

Youth obesity has a strong predictive value of tracking into adult obesity.^2^^,^^3^ Prevention of obesity in children is one critical way to help reduce rates of adult obesity and its associated chronic health conditions. However, there are few effective screening tools for identifying young children at risk for developing obesity, especially those in the healthy weight BMI category. Bruch (in 1939) made the early observation that children with obesity exhibit rapid growth velocity and tend to be taller than peers that do not exhibit excess weight.^57^ Many others have since confirmed the characteristic tall-for-age growth pattern in childhood obesity.^29^, ^30^, ^31^, ^32^, ^33^, ^34^ However, there has been less focus on the growth profiles of children in the healthy BMI weight category. On the basis of our evidence from mice that exhibit growth acceleration with longer bones before developing obesity when fed a high-fat diet,^35^ we sought to determine whether tall stature could be similarly used as an early indicator of obesity risk in a sample of Appalachian school children.

Our data support the hypothesis that height percentiles in kindergarten are associated with rapid BMI trajectories and later development of overweight or obesity by second or fifth grade. In our subset analysis of children in the healthy-weight BMI category, our data further show that kindergarteners who were tall for their age, yet still considered to have a healthy weight on the basis of BMI, had 3 times the odds of later developing overweight or obesity relative to children in lower height deciles. The findings that kindergarteners who were tall but with healthy weight developed overweight or obesity within a short 2- to 5-year period is consistent with early growth acceleration exhibited in a mouse model,^35^ and which has been described in the pediatric literature.^32^^,^^36^, ^37^, ^38^, ^39^, ^40^, ^41^^,^^58^

The key difference between our study and those that have previously reported an association with childhood height and later obesity is the young age at which we identified the pattern. For example, Stovitz et al found that children of normal weight who were tall relative to their peers in the third grade were more likely to become overweight or obese by the 12th grade.^59^ Our data show that the link between early tall stature and later obesity is evident even years earlier by kindergarten, suggesting that height could be used to assess obesity risk when children are just beginning elementary school. In addition, our results that kindergarten height is associated with a brief 2- to 5-year progression to overweight or obesity support previous studies that early childhood growth is a highly sensitive period for the detection and prevention of obesity.^21^^,^^60^, ^61^, ^62^, ^63^ Screening for obesity risk on the basis of height during the preschool years could offer even earlier opportunities for counseling and interventions than otherwise waiting for an obesity diagnosis to act.

Fifteen years ago, the US Preventative Services Task Force recommended using BMI to screen for obesity in children and adolescents between the ages of 6 to 18 years.^64^ Although an Expert Committee recommended that screening begin at age 2,^65^ the US Preventative Services Task Force did not find evidence to support screening children younger than 6 years of age at that time. However, new guidelines released by the American Academy of Pediatrics in 2023 highlight the importance of early evaluation and early intervention for mitigating the progression of obesity-related diseases, including the recommendation to annually screen children for overweight or obesity beginning at 2 years of age.^15^ On the basis of data presented here, we believe that rapid growth velocity could be the needed tool to identify children at risk for developing overweight or obesity potentially years before they would be detected using BMI.

Although it is difficult to identify a specific height cut-off for increased obesity risk, our data suggest that children in the top 3 height deciles (70th height percentile and above) merit closer scrutiny. On the basis of the lower confidence limit of the ORs shown in Figure 2, children in the top 3 height deciles have at least 2 times the odds of developing overweight or obesity compared with those in the lower height deciles. Awareness of such heightened risk for practitioners could prompt careful follow-up, including family counseling regarding diet, physical activity, and avoidance of sugar-sweetened beverages that would in theory be provided to all families. From a clinical care perspective, the key action to emphasize would be to get those children back for early and frequent follow-up aimed at obesity prevention rather than delaying treatment until obesity becomes apparent.

Taken together, our results suggest that tall stature alone may be a more effective indicator of obesity risk when de-coupled from BMI, especially after other factors such as genetic height potential^66^^,^^67^ and parent weight status and other contributing variables are considered.^49^^,^^68^, ^69^, ^70^ Others have previously detailed limitations of using BMI to flag children for obesity and health risks,^1^^,^^71^^,^^72^ including the positive relationship between excess height and excess adiposity.^73^ Because children will have already exhibited excess adiposity by the time their BMI approaches the overweight threshold, their trajectory toward obesity may be more difficult to reverse.^6^^,^^19^^,^^20^^,^^23^ For example, our subset analysis of children who already had an overweight or obesity BMI in kindergarten revealed that most (>84%) maintained that overweight or obesity status in second or fifth grade. Although our follow-up occurred in a shorter time frame, our findings are consistent with those from a national longitudinal study on entrenched obesity, which described the long-term (through age 14 years) persistence of obesity in 72% of children who had an obesity BMI when they entered kindergarten.^74^ Early risk recognition using tall stature could thus be one critical step in preventing chronic obesity.

### Adiposity Rebound and Age at Obesity Prediction

The age in which children were first examined in our study (average 5.9 years) marks the beginning of major changes in a child's social, hormonal, cognitive, and physical development as they experience adrenarche and transition from early to middle childhood.^75^ It is also the age in which adiposity shows a normal increase. “Adiposity rebound”^76^ refers to an increase in BMI that typically occurs in children at approximately 5-6 years of age after a natural decline in adiposity after the first year of life. Research has shown that the timing of adiposity rebound can be linked to the development of obesity and other chronic health complications. Early adiposity rebound that occurs before age 5 years is strongly linked to the later development of obesity, polycystic ovary syndrome, and other cardiometabolic abnormalities,^77^, ^78^, ^79^ suggesting that early timing of BMI increase could be used to predict childhood obesity. It is not surprising that our data demonstrate that tall stature at this critical age is also linked to the later development of obesity.

Interestingly, however, the early childhood increase in BMI is not universal to all human populations. Contemporary hunter-gatherers, whose diet and environment reflect a fundamentally different lifestyle compared with a US reference population (breastfed until 2-3 years age, high levels of physical activity, foraging diets), do not exhibit adiposity rebound at 5-6 years and only accumulate fat later in development after they have achieved maximum height velocity (10-14 years age) and made gains in skeletal growth.^80^ These data suggest that human growth is a highly plastic trait that can be modulated by diet and lifestyle, underscoring the importance of early obesity detection and prevention efforts during this critical growth window around 5-6 years of age.^74^^,^^81^

### Public Health and Clinical Implications

Public health and clinical initiatives seeking to expand childhood obesity—prevention programs could thus greatly benefit from considering tall stature and rapid growth velocity in preschool and early childhood as potential indicators of obesity risk, even (and especially) if children fit within a normal healthy BMI category. We believe that incorporating height, something already measured during well-child visits, could greatly strengthen the approach for combating the obesity epidemic by identifying at-risk children sooner and thereby creating an earlier window for actionable obesity prevention and intervention strategies (ie, Muñoz-Urtubia et al^82^ and Nicholson et al^83^) to reduce the burden of lifetime comorbidities. This is particularly relevant in rural areas of Appalachia, which have some of the greatest obesity rates and lowest health care outcomes in the country.^84^, ^85^, ^86^

### Strengths and Limitations

This study has several strengths and limitations that should be noted. Specifically, this is a large study, representative of West Virginia school children aged 5-11 years, with data collection spanning nearly 20 years between 1998 and 2017. The analysis incorporated several innovative methodologies, including SOUNDEX technology for merging the cross-sectional data into a longitudinal database and best linear unbiased predictors for individual BMI trajectories over time. Another notable strength is the early screening age and subsequent follow-up within 2 to 5 years. This unique study design enabled us to track the early co-occurrence of tall stature with the progression to overweight or obesity over a short time interval in young children just starting school. However, there are also limitations. Less than one-half of the 21 196 kindergarteners measured at initial screening had at least 1 match in second or fifth grade, limiting the final dataset to 9059. There were also no exclusion criteria for family history of endocrine disorders because these data were not collected on the screening form. The focus of the initial WV CARDIAC Project was only on screening for cardiovascular disease risk, and so we had no way to identify or exclude individuals with a family history of Cushing syndrome,^87^ polycystic ovary syndrome,^77^ or other endocrine disorders,^88^^,^^89^ which can alter bone growth as well as increase the risk of developing obesity.

In addition, because the focus of epidemiologic screening was on youth in West Virginia, a state with disproportionately high obesity rates,^85^ these results may not be as generalizable to other geographic regions with lower rates of youth obesity. Moreover, as the sample here was primarily White, this study needs to be replicated in regions with more diverse ethnic groups that have a greater obesity prevalence.^90^^,^^91^ Nevertheless, we believe that the greater incidence of obesity in our region provides a unique opportunity to study the link between early tall stature and later overweight/obesity that can be widely applicable for obesity risk detection among the general population.

## Conclusions

In conclusion, our longitudinal data spanning kindergarten to fifth grade demonstrate that increased height in kindergarten is associated with later development of overweight or obesity, even after controlling for BMI, age, sex, and race. These results support previous laboratory and cohort studies that suggest early childhood growth is a highly sensitive period for obesity detection and prevention. Tools to screen for obesity risk in young healthy weight children are lacking. Our data contribute meaningfully to this problem by demonstrating that height could be used to screen for obesity risk in healthy weight children as early as kindergarten. We suggest that height percentile could be a simple screening tool for obesity risk detection that has strong clinical and public health implications because it would enable earlier interventions for preventing youth obesity and its associated stigma and long-term health complications.

## CRediT authorship contribution statement

**Maria A. Serrat:** Writing – review & editing, Writing – original draft, Validation, Methodology, Investigation, Conceptualization. **Eloise Elliott:** Writing – review & editing, Supervision, Resources, Project administration, Methodology, Funding acquisition. **Lee A. Pyles:** Writing – review & editing, Supervision, Resources, Project administration, Methodology, Funding acquisition. **Christa L. Lilly:** Writing – review & editing, Writing – original draft, Validation, Software, Methodology, Investigation, Formal analysis, Data curation, Conceptualization.

## Declaration of Competing Interest

The CARDIAC Project was funded by many sources over the years, including the WV Bureau for Public Health, the 10.13039/100014150Benedum Foundation, the 10.13039/100000030Centers for Disease Control and Prevention, and the 10.13039/100000867Robert Wood Johnson Foundation. This study was also supported at Marshall University by resources from the 10.13039/100000002National Institutes of Health through the WV-CTSI (NIGMS award 2U54GM104942), WV-INBRE (NIGMS award P20GM103434), and Marshall University COBRE (NIGMS award P20GM121299). The study sponsors had no role in the study design, collection/analysis/interpretation of data, nor in writing the report or decision to publish. The authors declare no conflict of interest.
